# Environmental technology, economic complexity, renewable electricity, environmental taxes and CO2 emissions: Implications for low-carbon future in G-10 bloc

**DOI:** 10.1016/j.heliyon.2023.e16457

**Published:** 2023-05-22

**Authors:** Najia Saqib, Magdalena Radulescu, Muhammad Usman, Daniel Balsalobre-Lorente, Teodor Cilan

**Affiliations:** a Department of Finance, College of Business Administration, Prince Sultan University, Riyadh, Saudi Arabia; b Department of Finance, Accounting and Economics, University of Pitesti, 110040, Pitesti, Romania; c Institute for Doctoral and Post-Doctoral Studies, University “Lucian Blaga” Sibiu, Bd. Victoriei, No.10, Sibiu, Romania; d China Institute of Development Strategy and Planning, And Center for Industrial Economics, Wuhan University, Wuhan, 430072, China; e Department of Applied Economics I, University of Castilla-La Mancha, 16002, Cuenca, Spain; f Department of Management, Faculty of Economics and Management, Czech University of Life Sciences Prague, 16500, Prague, Czech Republic; g Department of Applied Economics, University of Alicante, Spain; h Department of Economics, University Aurel Vlaicu of Arad, Arad, Romania

**Keywords:** Energy productivity, Renewable electricity, Economic complexity, SDGs, G-10 countries, Environmental taxes

## Abstract

This study investigates the impact of environmental technological innovation, economic complexity, energy productivity, the use of renewable electricity generation, and environmental taxes on carbon dioxide (CO_2_) emissions in the G-10 countries for the timeframe from 1995 to 2020. The purpose of the study is to examine the need for a clear plan or strategy to achieve environmental objectives in G-10 countries. In both short-term and long-term projections, the increased use of environment-based technology, economic complexity, and renewable electricity generation has a major positive impact on carbon emission reduction. Moreover, the results demonstrate both unidirectional and bidirectional causality from carbon emissions to renewable energy, electrical generation, and environment-based technologies, respectively. Based on the results, the study proposes a number of concrete policies, such as updating modernized tax systems, increasing tax collection, providing individuals with the means to finance the Sustainable Development Goals through incentive regulations, and making grants from international organizations and the private sector available to finance investments toward the Sustainable Development Goals (SDGs) and carbon neutrality environment targets. This is the study's most significant contribution in order to attain a sustainable and low-carbon future in the G-10 countries, which has policy implications for governments and policymakers.

## 1 Introduction

**Fig. 1:**
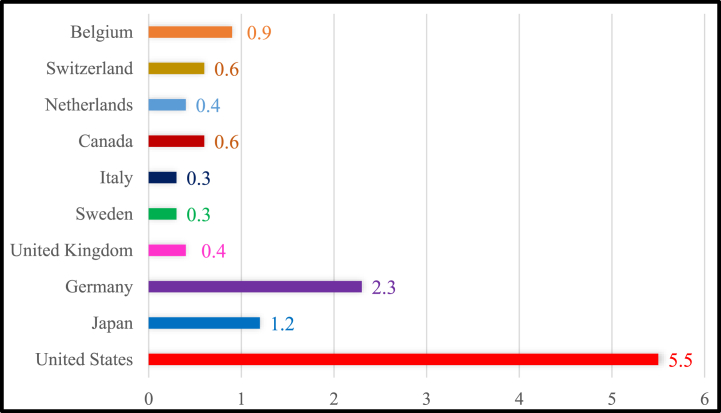
The amount of CO_2_ emissions in billion metric tons during 2018. Source: Global Economy [3].

The G-10 countries have committed to collaborating towards a future in which carbon emissions are drastically reduced and sustainable energy solutions predominate. Many renewable energy sources, such as solar, wind, water, and the earth's geothermal heat and power, have been widely adopted as means of achieving this objective. It is possible to come to the conclusion that countries' reliance on the generation of power from nonrenewable energy sources is significant. More encouragingly, the share of electricity produced from renewable sources has been growing over time [1]. Renewable energy generation (REG) is the production of electricity from renewable energy resources such as solar photovoltaics (PV), wind, geothermal, hydroelectric, and tidal. In spite of widespread knowledge that the earth's natural resources are finite, human beings continue to spend as if they were infinite. So, it is only natural that the planet will experience the consequences of global warming [2]. However, the efficiency of these initiatives is still up for question, and it's vital to evaluate the role other factors, such as environmental patents, economic complexity, energy productivity, and environmental taxes, play in bringing about reductions in carbon dioxide (CO_2_) emissions. Moreover, the G-10 countries are a group of the 10 largest economies in the world, and these countries emitted a total of 23.3 billion metric tons of CO_2_ emissions, which is equivalent to 55.3% of global carbon emissions. The United States is the largest emitter of CO_2_ emissions among the G10 countries, accounting for 14.6% of the total emissions, and Germany is the largest emitter of carbon dioxide among the European Union countries, with emissions of 2.3 billion metric tons. However, Canada is the country with the highest per capita carbon emissions among the G-10 countries, at 20 metric tons per capita, followed by the United States with 16.5 metric tons per capita. Moreover, the United Kingdom has reduced its carbon emissions by 42% since 1990, and is currently the leader among the G-10 countries in terms of reducing emissions in 2018 [3]. The G10 countries have all pledged to reduce their carbon emissions in order to combat climate change, with varying levels of ambition and progress. Fig. 1 presents the amount of carbon emissions in billion metric tons during 2018.

The global concern about climate change has led to a shift towards sustainable and low-carbon development in many countries [4,5]. The G-10 countries, comprising Belgium, Canada, Germany, Italy, Japan, Netherlands, Sweden, Switzerland, United Kingdom, and United States, have also recognized a dire need to reduce their pollution levels and transition towards more sustainable energy resources in the system. It is also observed that one of the key strategies adopted by these countries is the use of renewable energy to generate more electricity. Moreover, the economic complexity can be gauged by looking at the variety and sophistication of the products and services it offers to the global market. Economic complexity refers to the measure of the diversity and sophistication of a country's economy in terms of the range of industries it has, the level of technological advancement, and the level of human capital available. The higher the level of economic complexity, the more advanced and diversified the economy is. When a country's economy is large and varied, it can better weather shocks and accommodate renewable energy infrastructure [6,7]. While economic complexity is not a direct indicator of a country's progress towards developing sustainable energy systems, it does provide context for assessing the economic capabilities and innovation potential of a given country. The economies of the G-10 countries are highly diverse and technologically advanced, with a particular emphasis on service, high-tech, and knowledge-intensive industries, as well as the extraction and processing of natural resources. With a burgeoning renewable energy sector, investments in R&D for clean energy technology, and policies to encourage their adoption, these countries are well on their way to achieving their goal of reaching net-zero emissions by 2050 [8,9].

Renewable electricity production in G-10 countries is an important topic due to the need to mitigate carbon emissions and decrease reliance on non-renewable energy sources. These countries have established goals and developed strategies to expand their renewable electricity generation capacities. They are investing in wind, solar, hydroelectric, geothermal, biomass energy sources, and others. Some countries, such as Germany and Sweden, have set ambitious targets for achieving 100% renewable electricity generation in the future. The United States and Canada are also significant players in renewable electricity production, with hydroelectric power playing a significant role in both countries [10]. Overall, the G-10 countries are committed to increasing their renewable electricity generation capacities to mitigate climate change and reduce their dependence on non-renewable energy sources.

Environmental taxes, on the other hand, are a type of tax designed to discourage activities that harm the environment, such as pollution, carbon emissions, and waste disposal. These taxes are intended to incentivize individuals and businesses to engage in environmentally friendly practices and reduce their carbon footprint [11]. The impact of environmental taxes on the environment ultimately depends on a number of factors, such as the type of tax implemented, the level of enforcement and compliance, and the effectiveness of the incentives in driving behavior changes. Some studies suggest that environmental taxes can significantly reduce greenhouse gas emissions and other environmentally harmful practices, particularly when combined with other policy measures such as subsidies for renewable energy and investments in energy efficiency. However, the impact of environmental taxes on economic growth and competitiveness also needs to be considered. Some critics argue that higher taxes and regulations can impose excessive burdens on businesses, particularly in industries that are more dependent on fossil fuels, and may result in reduced economic growth and job losses [12].

With the G-10 countries' renewable energy consumption for power generation and the progress made towards sustainable energy infrastructure, the US is a leading producer and consumer of renewable energy. Wind, solar, and hydropower made up 12.2% of U.S. energy consumption in 2020. The country wants 100% clean energy by 2035. Hydropower, wind, solar, and geothermal are abundant in Canada. Canada's major energy supply was 18.9% renewable in 2019. The country wants net-zero emissions by 2050. The adoption of wind power in the UK has increased significantly. Renewables generated 43% of the nation's electricity in 2020. The UK targets net-zero emissions by 2050. France relies heavily on nuclear electricity, although it has also adopted renewable energy sources like wind and solar. Renewable energy generated 23.7% of the nation's electricity in 2020 [13]. Germany is a leader in solar and wind power adoption. Renewable energy generated 46.3% of the nation's electricity in 2020. The country wants 65% renewable electricity by 2030. Italy has advanced solar power adoption. Renewable energy generated 19.3% of the nation's electricity in 2020. Japan has advanced in the adoption of solar power. Renewable energy generated 8.8% of the nation's electricity in 2020. The country's target is around 22–24% renewable energy by 2030. Wind power has advanced in the Netherlands. 12.5% of the nation's electricity was renewable in 2020. The country wants 100% renewable electricity by 2050. Hydroelectric and wind power have been widely adopted in Sweden. Renewable energy generated 58% of the nation's electricity in 2020. The country wants net-zero emissions by 2045. Switzerland has advanced hydropower adoption. Renewable energy generated 63.7% of electricity in 2020. The country wants net-zero emissions by 2050. In essence, the G-10 nations have made great strides towards using renewable energy and decreasing their CO_2_ emissions. Still, they have a long way to go before they have a truly sustainable energy infrastructure. Overall, it is essential to strike a balance between promoting energy production and protecting the environment to ensure sustainable development. This requires careful consideration of the potential trade-offs between economic and environmental objectives and the implementation of well-designed policy measures that consider the unique circumstances and challenges faced by individual countries and businesses.

After debating all these facts and figures, there is a dire requirement that whether is there any significant effect of economic complexity on environmental degradation? Second, how does environmental technology protect the environment when it has the ability to tackle many environmental issues? And finally, what role of electricity production from renewable sources play in mitigating CO2 emissions in the G-10 countries? To tackle these concerns, policymakers and researchers are increasingly focusing their attention on comprehending energy through measurements such as productivity, intensity, and efficiency in order to confront the interrelated concerns of economic growth, the environment, and energy security [14,15]. Although efficiency is most commonly employed, energy productivity may provide a better path forward for a number of reasons. Thus, this paper examines the impact on energy productivity by studying its effects and connection to CO_2_ emissions. The conventional set of criteria for economic rewards described by Ref. [16] makes it more difficult for people to accept the idea that energy productivity is evaluated as GDP per unit of energy in dollars. As a result of its influence on energy productivity and costs, it also plays a significant role in determining environmental quality. Because of this, it has received a lot of attention for its role in achieving sustainable development and improving ecological integrity [17]. Increasing energy production has a multiplicative effect on economic growth because, first, it reduces the amount of energy needed for production while simultaneously lowering energy costs. Second, it lessens the need for foreign fossil fuels to generate electricity, reducing the overall impact on the environment [18].

Furthermore, it is anticipated that the results of this study will provide nations with additional assistance in reaching the Sustainable Development Goals (SDGs), particularly SDGs 7, 9, 11, 12, 13, and 15. Access to reliable, contemporary, low-cost, and sustainable energy sources is one of the aims of SDG-7 [19,20]. The importance of SDG-9's emphasis on sustainable manufacturing through technological innovation has grown in tackling the enormous ecological effect caused by the usage of fossil fuels for economic development [21]. Innovation has the potential to cut carbon emissions and promote the transition to energy efficiency and renewable energy sources, all of which are vital to generating increased productivity and economic growth [22]. Through SDG-11, the UN intends to improve urban areas in terms of diversity, safety, resilience, and sustainability [23]. Careful production and consumption practices are emphasized in SDG-12 in an effort to reduce strain on natural resources and reduce emissions [21]. One important part of this objective is encouraging sustainable ways of living and doing business. SDG-13 of the SDGs [24,25] emphasizes achieving net-zero carbon emissions by 2050. Sustainable Development Goal 15 (SDG-15) aims to preserve, restore, and encourage the use of terrestrial ecosystems in a way that is both environmentally and socially sustainable [26].

Environmental taxes have the potential to play a significant role in helping G-10 countries better protect their natural resources. By way of an environmental tax, people and the institutions they are associated with are required by law to take action to lessen their harmful effects on the environment. Greenhouse gas emissions, pollution levels in the air and water, and waste management are all examples of environmental concerns that could be addressed by regulation [27]. There are government entities in each of the G-10 countries tasked with enforcing environmental regulations. In the United States, this would be the Environmental Protection Agency, while in the United Kingdom, it would be the Environment Agency. Taxes on the ecosystem are fees placed on goods and services that have an adverse effect on the natural environment. The goal of these levies is to offer a financial incentive for individuals and corporations to take steps to lessen their negative effects on the environment. By establishing a transparent benchmark for environmental performance and holding firms and individuals accountable for their impact on the environment, environmental taxes have the potential to significantly enhance environmental quality in G-10 countries. In addition to generating cash for the government to use in funding environmental projects, environmental taxes can serve as a financial incentive for individuals and corporations to lessen their environmental impact. Investment tax credits, production tax credits, Conto Energia schemes, feed-in tariffs, SDE + schemes, and electricity certificate systems are just some of the G-10 countries' financial incentives for the development and deployment of renewable energy [28]. Renewable energy systems and other environmentally friendly innovations are protected by patents available from the Patent and Trademark Offices of these countries [29]. Patent protection for environmental innovations and a wide range of financial incentives are both available in the G-10 countries. Although environmental patents and taxes play a role, the adoption of sustainable energy systems is also driven by other factors such as government policies, technological advances, and public awareness and support.

This study is the first to use environmental patents, economic complexity, energy productivity, renewable energy, and environmental taxes to reduce CO_2_ emissions in the G-10 countries. Environmental patents cover carbon-reducing methods. Yet, economic complexity indicates a country's economic diversification, which may affect its ability to switch to renewable energy. Energy productivity measures energy efficiency and may affect renewable energy adoption. Environmental tariffs also encourage carbon reduction. Two ways this study contributes to the literature are: The literature gains empirical and methodological value from it. Our research uses G-10 country annual frequency data from 1995 to 2020. Moreover, this research paper aims to explore the policies and initiatives adopted by the G-10 countries to increase the use of renewable energy for electricity generation and reduce their carbon emissions. Specifically, this paper will focus on the interplay between renewable electricity generation, economic complexity, energy productivity, environmental taxes, and environmental technology in reducing carbon emissions. Several G-10 studies have employed panel causality tests to evaluate the relationship between variables of interest. This study is the first to examine environmental patents, economic complexity, energy productivity, renewable energy electricity generation, and environmental taxes on CO_2_ emissions reduction in the G-10 countries. This research will reveal the best G-10 policies and tactics for achieving their sustainable and low carbon future targets. The second-generation panel data analyses for cross-sectional dependence (CD) are more accurate and reliable than older methods. First-generation CD tests assume that dependence stays the same across all observations. Because of this, they can't show how CD changes over time. Second-generation tests allow for temporal and space-dependent dependence. Second-generation tests additionally evaluate the possibility of common factors, which are commonly present in panel data and drive cross-sectional dependency. Finally, second-generation tests identify cross-sectional dependence better and have improved finite-sample characteristics. The Cross-Sectionally Augmented Autoregressive Distributed Lag (*C*S-ARDL) test is novel because it accounts for cross-sectional dependence and heterogeneity, which are common in panel data, and produces more accurate and robust results. This test accounts for cross-sectional dependence by considering variable averages. This is one way to mitigate cross-sectional dependence-induced false regression. With panel data, where variable associations may vary among countries or blocs, this test allows for heterogeneous coefficients across cross-sections. This test feature improves cointegration relationship inference. This test is also flexible enough to handle nonlinearities and asymmetric effects in the cointegration relationship, unlike many panel cointegration tests.

The remainder of this study is set out as follows: Section 2 of this study is devoted to a survey of relevant previous research. Theory, data, and model specifications are presented in Section 3. Preliminary analysis and long-term estimation methods are discussed in Section 4. Thereafter, the results and discussions are presented in Section 5, and the study is wrapped up with some policy recommendations in Section 6.

## 2 Literature review

Spending on research and development activities is frequently used as a proxy for innovation in studies that attempt to quantify its environmental impacts. While some macro-level studies have produced conflicting results [29,30], others have found conflicting results for different time periods and different countries [31], and still others [32] have found that investments in R&D lead to lower levels of environmental pollution. Long-term ecological preeminence growth from green technological advancements is halted, as shown by this study's findings that the CO_2_-lowering effect depends on country-specific factors and that this impact usually recovers [[33], [34], [35]].

Scholars are trying to figure out how green technology will affect CO_2_ emissions around the world. Finding the tipping point in household income is essential for green technology to begin meaningfully reducing CO_2_ emissions. According to some earlier studies [29,36,37] the substantial costs associated with propagating new green technology provide a considerable economic hurdle. This is especially true for people who reside in developing nations. This finding is supported by the literature [38], which shows that businesses in developing nations cannot manage the large initial fixed costs involved with developing green technologies. In recent years, research and documentation have accumulated on carbon capture systems [39]. Moreover [40], investigated a link between industry value added, population density, foreign investment, renewable energy, and GDP growth on CO_2_ emissions in Central Asia and Europe. The findings explore that renewable energy and industry value added have an unfavorable influence on carbon emissions, pointing out that their growing use augments environmental pollution.

To help lower carbon emissions, some studies compare ecological patents with alternative energy technologies [41]. According to Ref. [42] analyzed the ICT sector expansion, energy transition, environmental regulations, and renewable energy in G-10 nations from 1990 to 2019. The outcomes display that ICT growth, environmental regulations and renewable energy adversely affect material footprints in the long- and short-run, signifying a considerable role in mitigating resource diminution. Some evidence suggests that CO_2_ emissions may encourage the registration of green patents and the funding of research and development [43]. According to Ref. [44] conducted a more in-depth analysis of the impact of economic complexity on greenhouse gas emissions for 25 European nations. The panel's empirical analysis, suggested that complex economic structures and energy usage patterns considerably influence carbon dioxide emissions. The study concluded that as economic complexity and the use of nonrenewable energy sources increase, so does the risk of causing environmental damage. Sixteen European countries' ecological footprints were analyzed by Ref. [45] to learn more about the connections between trade policy, economic growth, and energy use. The results of the ARDL panel showed that the use of nonrenewable energy and economic expansion had both a very damaging environmental impact, although the use of renewable energy had a tendency to lessen this impact. According to Ref. [46] proposed that the economic complexity of European countries has U-shaped implications for their CO_2_ emissions. The findings demonstrated, from a critical standpoint, that the complexity of exports increases pollution in the beginning, but then, after a certain point, economic complexity reduces pollution. The effects of environmental policies on countries with low, middle, and high incomes were examined by Ref. [47]. From the data, it appears that energy market, economic sector, and habitat shocks have short-term effects on high-income and middle-income countries' CO_2_ emissions, after which the trend may resume. To be more specific, the study concluded that governments should prioritize long-term environmental measures above establishing targets that aren't necessary owing to shocks in CO_2_ emissions. Moreover [48], analyzed the dynamic nexus between human capital through labor-added technology, economic growth, and CO_2_ emissions in Pakistan from 1975 to 2020. The findings reveal that an increase in human capital will reduce the pressure on the environment in Pakistan. Similarly, R&D spending and labor-augmented technology increase the environmental quality. According to Ref. [44] have done the most research on the economic complexity of European countries, and it is justified to examine how structural change in a complex economic system relates to fossil fuels and environmental impact.

The energy-CO_2_ emission nexus has generated a pile of research [[49], [50], [51]]. There is a lack of agreement on whether or not energy consumption directly causes CO_2_ emissions. Some show unidirectional causation, some show bidirectionality, and still others show that there is no causal connection. Several studies challenge unidirectional causality by showing a link between energy use and CO_2_ emissions, while others find the exact opposite to be true. Remarkably, different findings were found from studies conducted on the correlation between the two characteristics in different countries. The direction of causation (whether one variable results from the other) is only part of the puzzle when trying to establish a link between energy consumption and carbon dioxide emissions [52]. This causality has been established using a number of procedures and variables; naturally, each of them has yielded a unique finding, which is somewhat owing to the diverse methodologies employed. Simply put, if there is just one direction of dependence between the two variables, as such, the two variables depend on one another in a reciprocal fashion, indicating a bidirectional causality link. Utilizing the panel vector error correction model [53], found that while there is no long-term causal link between renewable energy consumption and CO_2_ emissions, there is a short-term association running in the opposite direction, from renewable energy to CO_2_ emissions. As a result, while renewable energy and carbon dioxide emissions are independent in the long run, carbon dioxide emissions are dependent on renewable energy in the short run [54,55]. Since more people are becoming aware of how important it is to look at energy sources in terms of emissions, there has been a significant development in the literature to capture emissions from energy sources. Recent energy research indicates that reducing CO_2_ emissions is essential due to the implications that they have on global warming [56,57]. The impacts of renewable and fossil fuel energy use on panel data of countries' carbon impacts were studied by Ref. [58]. It is challenging to halt global warming since CO_2_ emissions remain in the atmosphere for 50–100 years. Switching from electric power facilities that rely on fossil fuels to those that rely on renewable energy sources is one way to cut down on carbon dioxide emissions. The energy sector does not appear to be the primary source of CO_2_ emissions, as the positive and negative effects of CO_2_ emissions on renewable energy sources indicate. Moreover, the government of each country should increase the percentage of renewable energy in its portfolio since these sources generate electricity without releasing CO_2_ emissions.

The results of the panel cointegration and regression analyses showed that the environment is being severely harmed in ASEAN economies as a result of the use of nonrenewable energy. The findings indicate that climate change and cleaner manufacturing are likely to be harmed as a result of the ASEAN region's growing economy. According to Ref. [59] examined the same variables as shown by the results of the panel estimators and claimed that using renewable energy helped the environment, while using nonrenewable energy was shown to worsen it. As a rule of thumb, energy usage is one of the most important factors in calculating an organization's impact on the environment. Yet, most research focuses on non-renewables and the impact renewable energy has on the environment. While the impact of fossil fuels and a complex economy on environmental quality has not been fully investigated, much work remains to be done.

The results of prior studies show that environmental regulations enhance environmental quality. Environmental regulations are said to lessen carbon dioxide emissions in the BRICS countries [19,60]. Carbon pricing is essential for reducing carbon dioxide emissions, and environmental regulations have been shown to enhance the environment in OECD countries [61,62]. In China, researchers discovered consistent outcomes [63,64]. Research suggests that China's environmental difficulties could be mitigated by implementing both economic and non-economic reforms to the country's environmental policies [65,66]. Environmental regulations tend to have negative effects on environmental quality in low- and middle-income nations while having positive effects on environmental quality in high-income countries, according to a study of 117 countries [67]. This article contends that tight environmental regulations in developed G-10 countries force polluting commodity industries to relocate to countries with lax environmental regulations. Because of this, rich nations funnel FDI that causes pollution to countries with lax environmental regulations. Environmental quality is directly affected by ecological regulations enforcement, while other significant macroeconomic attributes that are related to environmental quality are indirectly affected. One way that environmental regulations can help make the world a better place to live is by increasing macroeconomic aggregates like economic growth, energy consumption, human capital, and trade-openness. Environmental regulations are not particularly good at ensuring ecological development at first, but they do so over time and in a way that improves environmental quality [68]. While environmental regulations effects on CO_2_ emissions have been the subject of numerous studies, their global effects on carbon emissions have received considerably less attention.

Triangulating our debate from the abovementioned literature, the authors conclude that there are two main research gaps that this study needs to tackle. (i) There are negligible findings that examine patent application, economic complexity, energy productivity, renewable electricity production, environmental tax, and carbon emissions from the sunshade of dynamic estimation. (ii) There are no of the single indications in the existing body of literature that properly assimilate diverse SDGs to advocate policy-level assessments. In that regard, there are particularly few existing studies that have addressed the link between the selected variables and SDGs at the policy echelon, in that regard, as long as the prospect exists to tackle the same in this study. This provides a panorama for researchers to achieve parallel research in other emerging and developed economies and present resonance policy options towards the interface of patent application, economic complexity, energy productivity, renewable electricity production, environmental tax, and CO_2_ emissions. In this view, the anticipated analysis under this research would support the central governing legislative body and ecological activists, particularly in the declared G-10 economies. As a result, the present research follows the objective of linking this gap by spotlighting G-10 economies as a new deliberation, technological innovations, and energy use as means for humanizing ecological contamination in the course of reduced CO_2_ emissions. The proposed hypotheses can be summarized as follows.

Hypothesis 1 (H1): Environmental technology is a key factor in reducing environmental pollution in G-10 countries.

Hypothesis 2 (H2): Economic complexity augments CO_2_ emissions in G-10 countries.

Hypothesis 3 (H3): Energy productivity increases the pollution level in G-10 countries.

Hypothesis 4 (H4): Renewable electricity generation reduces the dependence on fossil fuels and thereby reduces environmental pollution in G-10 countries.

Hypothesis 5 (H5): Environmental taxes can incentivize companies to reduce CO_2_ emissions in G-10 countries.

## 3 Theoretical modelling

Environmental technology plays a crucial role in reducing environmental pollution, especially through the use of renewable electricity. However, a number of factors, such as economic complexity and a lack of appropriate environmental tax policies, can make the adoption and implementation of these technologies more difficult. In this pursuit, environmental technology and renewable electricity can provide sustainable solutions to address the challenge of environmental pollution, but these technologies can be prohibitively expensive or require significant technical expertise. For this reason, it may take time for these technologies to be adopted and integrated into existing systems, and the cost of adoption may be a significant barrier. However, economic complexity can play a role in the adoption of environmental technologies [11]. Those communities or countries with more diversified economic activities are more likely to adapt and adopt these technologies since their economies are better equipped to absorb the costs of investments in environmental technology than those with less complex economies [7]. The extent to which renewable electricity is adopted is influenced by economic factors such as the availability and cost of alternative energy sources as well as policy incentives such as subsidies and tax credits [4]. Furthermore, environmental tax policies are critical to promoting the adoption of environmentally sound technologies. Environmental taxes can act as incentives to encourage the adoption of renewable electricity and other alternative energy sources by making environmentally harmful activities, such as fossil fuel exploitation, more expensive or less desirable. These taxes can also help mitigate the negative effects of environmental pollution and encourage a shift toward more sustainable practices. The level of environmental tax is determined by the government and is typically based on the amount of pollution emitted by a company. The revenue generated from environmental taxes can be used to fund investments in environmental technology or to provide incentives for companies to adopt renewable electricity generation. In general, the integration of environmental technology, economic complexity, environmental taxes, and renewable electricity is a complex process that requires a multi-faceted approach. However, the effectiveness of these tools is influenced by economic factors such as the level of economic complexity and the availability and cost of alternative energy sources. Policymakers must carefully balance these factors in order to create effective policies for reducing environmental pollution. By developing and implementing appropriate policies and technologies, we can reduce environmental pollution and create a more sustainable future.

## 4 Model specification and methodology

### 4.1 Data

This study will make use of G-10 countries' annual panel data from 1995 to 2020. Based on available information, we have chosen to focus on the following countries: Belgium, Canada, Germany, Italy, Japan, the Netherlands, Sweden, Switzerland, the United Kingdom, and the United States. The variables, units of measure, and data sources are listed in Table 1.

**Table 1:** Variables, measurement unit and data sources (1995–2020).

Variables	Symbol	Measurement unit	Data Sources
CO_2_ emissions	CO_2_	Million tons per capita	WDI
Patents application	TEC	Total number	WDI
Economic complexity	ECI	Index	OECD
Energy productivity	ENP	Percentage of total primary energy supply	OECD
Renewable electricity production	REG	Total gigawatt hours	OECD
Environmental tax	ETX	% of total patents on environment technologies	OECD

### 4.2 Model

The following is a presentation of the mathematical equation of the econometric function presented in Equation (1) as follows:

(1) $${CO}_{2,it}=\beta_{0}{+\beta }_{1}{TEC}_{it}{+\beta }_{2}{ECI}_{it}+{\beta_{3}ENP}_{it}+{{+\beta }_{4}{REG}_{it}+\beta }_{5}{ETX}_{it}+E_{it}$$

Where CO_2_, TEC, ECI, ENP, REG, and ETX represent, in that order, carbon dioxide emissions, the total number of environmental patent applications, the economic complexity index, energy productivity, the production of renewable electricity, and environmental taxes, respectively. Where $E_{it}$ = error term, the countries are shown by the subscript (i = 1 ….n), and the time is stated by the subscript (t = 1 ….t). Equation (1), CO_2_, is a dependent variable, while TEC, ECI, ENP, EEG, and ETX are independent variables. To avoid data sharpness and heteroscedasticity, we convert all the data into natural logarithms [57,69].

Table 2 lists the descriptive data in brief the mean values of CO_2_, TEC, ECI, ENP, REG and ETX are 2.823, 10.157, 0.689, 1.927, 12.222, and 0.823, respectively. While the standard deviations of the CO_2_, TEC, ECI, ENP, REG, and ETX are 0.823, 2.198, 0.314, 0.999, 1.142, and 0.3921, respectively. Moreover, the Jarque-Bera statistic reported that all variables have statistical significance and reject the null hypothesis of data normality, except CO_2_. Furthermore, Table 2 also shows the correlation matrix, the range of correlation exists between −1 and +1; if the correlation coefficient is near −1, it reveals a strong negative correlation between the variables, otherwise a strong positive correlation between the variables.

**Table 2:** Descriptive statistics and pairwise correlation matrix.

Descriptive statistics								
Variables	Mean	Min.	Max.	Std. Dev.	Skew.	Kurt.	Jarque-Bera	Prob.
CO2	2.8230	1.2910	3.1900	0.8230	0.0800	2.6720	2.6340	0.0010
TEC	10.1570	5.7230	14.8730	2.1980	0.1830	1.8340	15.8390	0.0000
ECI	0.6890	−0.8450	1.1270	0.3140	−0.8060	3.1930	23.0170	0.0000
ENP	1.9270	−0.3020	3.7320	0.9990	−0.2810	2.3520	9.2710	0.0050
REG	12.2220	8.9650	16.5780	1.1420	0.5210	3.1980	13.8290	0.0000
ETX	0.8230	−0.5030	1.5210	0.3921	−0.5410	2.8470	13.9980	0.0000

Pairwise correlation matrix						
Variables	CO2	TEC	ECI	ENP	REG	TECH
CO2	1.0000					
TEC	0.7245*	1.0000				
ECI	0.4188*	0.6978*	1.0000			
ENP	0.4896*	0.7154*	0.5887*	1.0000		
REG	−0.3957*	−0.0978*	0.4152*	0.2174*	1.0000	
ETX	−0.4152*	−0.1814*	−0.3514*	−0.3677*	−0.1914*	1.0000

Note: * designates the significance level at 1%.

### 4.3 Methodology

#### 4.3.1 Cross-sectional dependence tests and slope homogeneity tests

Before testing with co-integration and stationarity of panel data, the cross-sectional dependence (CD) test is conducted to reduce the likelihood of obtaining misleading, contradictory, and otherwise wrong results [70]. This study makes use of the Pesaran [70] CD test, which is formulated as given in Eq. (2) as:

(2) $$CD=\sqrt{\frac{2X}{Y\left(Y-1\right)}}\left[\sum_{i=1}^{m-1}\sum_{k=i+1}^{m}{\hat{\varphi_{ik}}\varphi }_{ik}\right]$$

Where Y = 1, 2 …, N (year, 1995–2020) and $\varphi_{ik}^{t}$ shows pair-wise correlation coefficients. The following assumptions are used to determine the presence of CD. There is no CD in H_0_, but there is a CD in the alternative hypothesis. To conclude that CD is a component of the panel data, the null hypothesis is rejected when the p-value of the test is less than 0.05.

This study empirically investigates the slope homogeneity (*S*–H) test after the CD test. According to Ref. [71] created the *S*–H test to analyze if the co-integration coefficients are homogeneous or if the coefficients of the explanatory factors differ from one unit (country) to the next. Unlike traditional homogeneity evaluation methods, *S*–H is appropriate for panel data since it takes CD into account. The *S*–H test is mathematically expressed as Eqs. (3), (4)) [71] as:

(3) $${\tilde{\Delta }}_{,HT}={\left(N\right)}^{\frac{1}{2}}{\left(2k\right)}^{-\frac{1}{2}}\left(\frac{1}{N}\tilde{S}-k\right)$$

(4) $${\tilde{\Delta }}_{AHS}={\left(\frac{2k\backslash (T-k-1}{T+1}\right)}^{-\frac{1}{2}}\left(\frac{1}{N}\tilde{S}-2k\right)$$

Where $\left({\tilde{\Delta }}_{HT}\right)$ and $\left({\tilde{\Delta }}_{AHS}\right)$ represent the delta tilde and the adjusted delta tilde of the slope homogeneity, respectively.

#### 4.3.2 Unit root tests

Misconstrued inferences can be drawn from conventional unit root tests because they presume model cross-section independence [72,73]. CIPS and CADF both test for CD. For examples, see Eqs. (5), (6), (7)) as:

(5) $$\Delta Z_{i,t}=\omega_{i}+\omega_{i}W_{i,t-1}+\sum_{j=0}^{k}\omega_{ij}\Delta {\bar{Z}}_{i,t-1}+\sum_{j=1}^{k}\omega_{ij}\Delta Z_{i,t-1}+U_{i,t}$$

(6) $$CIPS=\frac{1}{T}\sum_{i=1}^{T}j_{i}\left(T,N\right)$$

(7) $$\hat{CIPS}=\frac{1}{T}\sum_{i=1}^{n}{CADF}_{i}$$

#### 4.3.3 Cointegration tests

This study utilized cointegration tests to investigate the long-run cointegration between the selected series in both categories [74]. The Westerlund cointegration method is an important breakthrough since it can be applied to slope heterogeneity models [75]. In addition, the test takes cross-sectional dependence into consideration. In Eqs. (8), (9), (10), (11)), the Westerlund test employs two-group test statistics (G_t_ and G_a_) and two panel statistics (P_t_ and P_a_).

(8) $$G_{\tau }=\frac{1}{N}\sum_{i=1}^{N}\frac{{\hat{\alpha }}_{i}}{SE\left({\hat{\alpha }}_{i}\right)}$$

(9) $$G_{\alpha }=\frac{1}{N}\sum_{i=1}^{N}\frac{T{\hat{\alpha }}_{i}}{{\hat{\alpha }}_{i}\left(1\right)}$$

(10) $$P_{\tau }=\frac{{\hat{\alpha }}_{i}}{SE\left({\hat{\alpha }}_{i}\right)}$$

(11) $$P_{\alpha }=T\hat{\alpha }$$

where, ${\hat{\alpha }}_{i}$ is shown by $SE\left({\hat{\alpha }}_{i}\right)$ as the standard random error. The semi-parametric kernel approach of ${\hat{\alpha }}_{i}\left(1\right)$ is ${\hat{\alpha }}_{i}\left(1\right)$.

#### 4.3.4 *C*S-ARDL estimates

Consider the conventional panel ARDL general form given in equation (12). Where ${{CO}_{2}}_{i,t}$ shows the dependent variable and the term ${{CO}_{2}}_{i,t-j}$ denotes lagged dependent variable, $Y_{i,t-j}$ is the vector of all explanatory variables. In the subscripts i and *t* stand for countries (1, 2 …., n) and time periods (1995–2020), respectively. The term $R_{i}$,; $\propto_{ij}$, $\emptyset_{ij}$ and $E_{it}$ represent the fixed effects, coefficient of the lagged regressor, m × 1 coefficient vectors (lagged regressors), and error term, respectively.

(12) $${{CO}_{2}}_{i,t}=\sum_{j=1}^{p}\propto_{ij}{{{CO}_{2}}_{i,t}}_{-j}+\sum_{j=0}^{q}\emptyset_{ij}Y_{i,t-j}+R_{i}+E_{it}$$

In the occurrence of CD, the conventional panel ARDL given in equation (12) results are biased [76]. To solve the issue of the CD, a different estimating approach known as the Cross-Sectionally Autoregressive Distributed Lag Model (*C*S-ARDL) is used. According to Ref. [77], the regressors' cross-sectional averages should be included as additional lags to the ARDL specification in equation (12). The modified Eq. (13), which includes the cross-sectional lag factor, is follows:

(13) $${{CO}_{2}}_{i,t}=\sum_{j=1}^{p}\propto_{ij}{CO}_{2\;i,t-j}+\sum_{j=0}^{q}\emptyset_{ij}Y_{i,t-j}+\sum_{j=0}^{r}\omega_{ij},I{\bar{N}}_{t-1}+R_{i}+E_{it}$$

Where in equation (13), ${\bar{N}}_{t-1}=\left({\bar{CO}}_{2,i,t-j},{\bar{Y}}_{i,t-j}\right)$ are the dependent variable and regressor averages. Moreover, *p, q,* and *r* represent cross-sectional average lag times and individual lag times for each variable. While $\bar{N}$ essentially depicts cross-section averages and eliminates cross-section dependencies [66,78]. The *C*S-ARDL approach's long-run coefficient estimations may be computed as in Eq. (14) as:

(14) $${\hat{\vartheta }}_{{CS-ARDL}_{i-j}}=\frac{\sum_{j=0}^{q}{\hat{\emptyset }}_{ij}}{1-\sum_{j=1}^{p}\hat{\delta_{ij}}}$$

In addition to this, Eq. (15) can be stated in error correction form as seen as:

(15) $${{\Delta CO}_{2}}_{i,t}=\Omega_{i,}\left[{CO}_{2\;i,t-j}-\lambda_{i,j}y_{i,t}\right]-\sum_{j=1}^{p-1}\delta_{ij}\Delta_{I}{CO}_{2\;i,t-1}+\sum_{j=0}^{q}\emptyset_{ij}{\Delta y}_{i,t}+\sum_{j=0}^{r}\beta_{i},I{\bar{N}}_{t-1}+R_{i}+E_{it}$$

Where $\Delta_{I}=t-\left(t-1\right)$.

#### 4.3.5 Robustness test

The study [79] designed the Fully Modified Least Square (FMOLS) to carry out an improved co-integrating regression estimation as presented in equation (16). The panel cointegration regression in this work employed the [80] heterogeneous FMOLS estimator because of its ability to account for endogeneity bias and serial correlation. The asymptotic distribution of the dynamic OLS estimator was the same as that of the panel FMOLS estimation. To verify the reliability of the result, we estimated DOLS and FMOLS in Eq. (16) as:

(16) $$\beta_{NT}^{*}-\beta =\left(\sum_{i=1}^{N}L_{i}^{-2}\sum_{i=1}^{T}{\left(\chi_{it}-{\bar{\chi }}_{it}\right)}^{2}\right)\sum_{i=1}^{N}L_{i}^{-1}L_{i}^{-1}\left(\sum_{i=1}^{T}\left(\chi_{it}-{\bar{\chi }}_{it}\right)\mu_{it}^{*}-T{\hat{\gamma }}_{i}\right)$$

Furthermore, the Augmented Mean Group (AMG) test, developed by Ref. [81] is utilized for the robustness test in this study. It has been shown that the AMG method is effective at addressing the CSD, non-stationarity, endogeneity, and slope variability of longitudinal data. First and second order AMG implication, written as Eqs. (17), (18)) are:

(17) $$\Delta X_{it}=\delta_{i}+\beta_{i}\Delta Y_{it}+\gamma_{i}A_{t}+\sum_{t=2}^{T}\delta_{i}\Delta D_{t}+\varepsilon_{it}$$

(18) $${\hat{\beta }}_{AMG}=N^{-1}\sum_{i=1}^{N}{\hat{\beta }}_{i}$$

#### 4.3.6 Panel granger causality test

The preceding *C*S-ARDL fails to explain the causal connection. The Panel causality, which is often employed [82] for determining whether there is a causative relationship between the variables and its presented from Eq. (19), (20), (21), (22), (23), (24), (25), (26), (27), (28)) as:

(19) $${\Delta CO}_{2,it}=\emptyset_{1,j}+\sum_{k}\emptyset_{11,ik}{\Delta CO}_{2,it-k}+\sum_{k}\emptyset_{12,ik}{\Delta TEC}_{it-k}+E_{1,it}$$

(20) $${\Delta TEC}_{it}=\emptyset_{2,j}+\sum_{k}\emptyset_{21,ik}{\Delta CO}_{2,it-k}+\sum_{k}\emptyset_{22,ik}{TEC}_{it-k}+E_{2,it}$$

(21) $${\Delta CO}_{2,it}=\emptyset_{1,j}+\sum_{k}\emptyset_{11,ik}{\Delta CO}_{2,it-k}+\sum_{k}\emptyset_{12,ik}{\Delta ECI}_{it-k}+E_{1,it}$$

(22) $${\Delta ECI}_{it}=\emptyset_{2,j}+\sum_{k}\emptyset_{21,ik}{\Delta CO}_{2,it-k}+\sum_{k}\emptyset_{22,ik}{ECI}_{it-k}+E_{2,it}$$

(23) $${\Delta CO}_{2,it}=\emptyset_{1,j}+\sum_{k}\emptyset_{11,ik}{\Delta CO}_{2,it-k}+\sum_{k}\emptyset_{12,ik}{\Delta ENP}_{it-k}+E_{1,it}$$

(24) $${\Delta ENP}_{it}=\emptyset_{2,j}+\sum_{k}\emptyset_{21,ik}{\Delta CO}_{2,it-k}+\sum_{k}\emptyset_{22,ik}{ENP}_{it-k}+E_{2,it}$$

(25) $${\Delta CO}_{2,it}=\emptyset_{1,j}+\sum_{k}\emptyset_{11,ik}{\Delta CO}_{2,it-k}+\sum_{k}\emptyset_{12,ik}{\Delta REG}_{it-k}+E_{1,it}$$

(26) $${\Delta REG}_{it}=\emptyset_{2,j}+\sum_{k}\emptyset_{21,ik}{\Delta CO}_{2,it-k}+\sum_{k}\emptyset_{22,ik}{REG}_{it-k}+E_{2,it}$$

(27) $${\Delta CO}_{2,it}=\emptyset_{1,j}+\sum_{k}\emptyset_{11,ik}{\Delta CO}_{2,it-k}+\sum_{k}\emptyset_{12,ik}{\Delta ETX}_{it-k}+E_{1,it}$$

(28) $${\Delta ETX}_{it}=\emptyset_{2,j}+\sum_{k}\emptyset_{21,ik}{\Delta CO}_{2,it-k}+\sum_{k}\emptyset_{22,ik}{ETX}_{it-k}+E_{2,it}$$

**Fig. 2:**
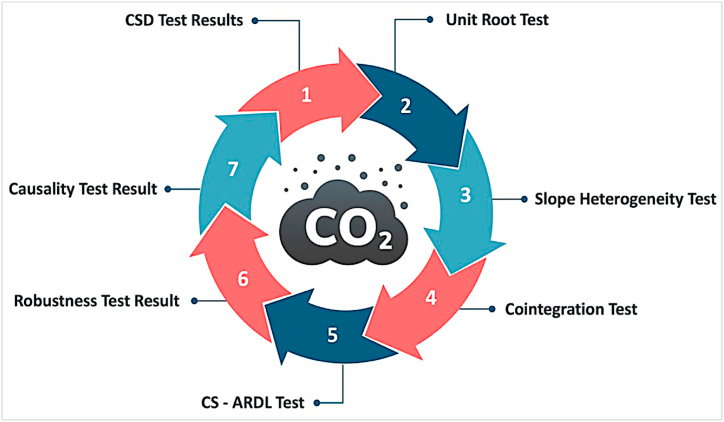
Methodology strategy.

The null hypothesis of the Granger causality test is that the lagged of the explanatory variable does not justify the variation in the dependent variable. So, therefore, $H_{0}:\emptyset_{12ik}=0$ or $H_{0}:\emptyset_{21ik}=0$. If the null hypothesis is rejected *TEC, ECI, ENP, REG,* and *ETX* cause CO_2_ emissions. This graphical presentation of methodological strategy is depicted in Fig. 2 as:

## 5 Results and discussion

In this study, we employed CD tests such as Breusch-Pagan LM, Pesaran scaled LM, Bias-corrected scaled LM, and Pesaran CSD to study the CD properties of the variables of interest. Table 3, shows the estimates of the CD test report that all statistics reject the null hypothesis of no cross-sectional dependency at the 1% level of significance.

**Table 3:** Cross-sectional dependence tests results.

Variables	Breusch-Pagan LM		Pesaran scaled LM		Bias-corrected scaled LM		Pesaran CSD	
	Stat.	Prob.	Stat.	Prob.	Stat.	Prob.	Stat.	Prob.
CO_2_	425.2514*	0.0000	51.2547*	0.0000	46.4832*	0.0000	15.2105*	0.0000
TEC	261.6758*	0.0000	27.8756*	0.0000	25.2446*	0.0000	10.2317*	0.0020
ECI	450.9851*	0.0000	51.8857*	0.0000	45.7201*	0.0000	13.0071*	0.0000
ENP	385.8544*	0.0000	49.8950*	0.0000	41.2423*	0.0000	9.8422*	0.0000
REG	315.1150*	0.0000	32.8700*	0.0000	27.2458*	0.0000	11.7812*	0.0000
ETX	350.5780*	0.0000	29.8223*	0.0000	23.7110*	0.0000	14.8782*	0.0000

Note: * designates the significance level at 1%.

In Table 4, the findings show the estimates of *S*–H and reported that there is heterogeneity issue at 1% level of significance.

**Table 4:** Slope-homogeneity test results.

${\tilde{\Delta }}_{HS}$		${\tilde{\Delta }}_{AHS}$	
Stat.	Prob.	Stat.	Prob.
17.46671*	0.0000	20.4666*	0.0000

Note: * designates the significance level at 1%.

Table 5 shows the results of second-generation unit root tests (i.e., CIPS and CADF). The variables ECI and ETX have been stationary at level in both tests, while CO_2_, TEC, ENP, and REG have not followed the stationary at level I (0). However, all the variables are following the stationarity property after taking their first integration order I (1).

**Table 5:** Unit-root test results.

Variables	CIPS		CADF	
	I (0)	I (1)	I (0)	I (1)
CO_2_	−0.1514	- 4.5241*	- 0.5854	- 4.6175*
TEC	−1.3089	−5.4987*	−1.8777	−5.9631*
ECI	−3.8754*	−3.5870*	- 4.6184*	- 4.5487*
ENP	−1.5087	−2.6703*	−1.5697	−3.3647**
REG	−1.4099	−5.7128*	−1.5403	−3.0372**
ETX	−3.2780	−5.4870*	- 4.5678*	−5.8942*

Note: * and ** designate the significance level at 1%, and 5% respectively.

Based on the results of the cointegration test, as shown in Table 6, there seems to be a link between the variables that lasts for a long time. The group and panel statistics that Westerlund put together show that this is accurate. Since the purpose of these tests is to engage with cross-sectional dependence in a panel study, there are many studies that have applied a similar approach [10,27,83,84].

**Table 6:** Cointegration test results.

Statistics	Values	Z-values	*P*-values	Robust *P*-values
Gτ	−7.8541*	−4.6184	0.0000	0.0000
Gα	−16.3321**	2.0005	0.0013	0.0000
Pτ	−18.8540*	−4.9781	0.0000	0.0000
Pα	−20.1151*	−2.2117	0.0012	0.0000

Note: * and ** designate the significance level at 1%, and 5% respectively.

The panel's *C*S-ARDL findings are shown in Table 7. In the long and short run TEC, ECI, ENP, REG, and ETX have a negative effect on CO_2_ emissions. The coefficient of TEC is −0.5845% for the long run and −0.0378% for short run, which means that a 1% increase in TEC is recorded to reduce CO_2_ emissions by 0.5845% in the long run and 0.0378% in the short-term estimates, assuming everything else remains constant. The results of the present investigation are in line with [85,86]. The results of the patents show that creating and funding inventions with an eye toward the environment may help cut down on unnecessary CO_2_ emissions. In addition, the results imply that the countries examined in this study can cut their CO_2_ emissions by funding the development of technology to fulfill their carbon-zero ambition. The empirical results also suggest that countries under investigation may be justified in lowering their barriers to environmentally productive technology if they grant patents related to the protection of the natural environment. On the other hand [57], benefiting the environment and the economy, as well as contributing to the achievement of SDG-9, can be achieved by switching to energy-efficient, cutting-edge manufacturing methods.

**Table 7:** *C*S-ARDL parameter estimates.

Variables	Long-run		Short-run	
	Coeff.	Prob.	Coeff.	Prob.
TEC	−0.5845**	0.0510	−0.0378**	0.0724
ECI	−0.3487*	0.0000	−0.8451*	0.0035
ENP	−0.7189*	0.0000	−0.0298**	0.0560
REG	−0.6920*	0.0000	−0.6471**	0.0542
ETX	−0.0547*	0.0002	−0.0785**	0.0110
ECM (−1)	–	–	−0.6178*	0.0001

Note: * and ** designate the significance level at 1%, and 5% respectively.

However, ECI is negative and statistically significant at the 1% level, with a reduction in CO_2_ emissions of 0.3487% in the long-run estimate and −0.8451% in the short-run estimate, everything else being equal. The study [87] investigated the relationship between CO_2_ and ECI in France. They reported that ECI reduces CO_2_ emissions. While [88] corroborated this finding and validated that ECI reduces emissions in high-income countries, there are also negative consequences of ECI on the environment in middle- and low-income countries. Finally, both studies reported that higher levels of ECI appear to be good for environmental quality. However [46], found that the most complex nations disputed the prior findings of [87,88], revealing that ECI accelerates environmental deterioration. On the contrary [89], revealed that ECI raises CO_2_ levels in the United States. Similarly [90], proposed that CO_2_ emissions rise when ECI increases in the seven countries with the greatest ECI. Furthermore [7], shown that collective economic complication reduces environmental degradation in India. Moreover [91], studied the relationship between economic complexity and CO_2_ emissions, and proposed that increasing economic complexity enhances environmental quality. Also [92], explored how economically complex counties, provinces, and large cities in Brazil affected environmental quality and discovered that more economic complexity is related to lower environmental deterioration.

The coefficient of ENP is −0.7189% in the long run and −0.0298% in the short run, indicating that a 1% increase in ENP is recorded to reduce CO_2_ emissions by 0.7189% and 0.0298% in the long and short-run estimations, respectively. Energy productivity is sometimes referred to as energy efficiency, and if a country's production system is efficient, it will require less energy for a high output, reducing carbon emissions [93]. Thus, energy production has an adverse effect on carbon emissions. Together with clean and environmentally acceptable energy sources, energy productivity is viewed as a vital factor in lowering fossil fuel use and reaching sustainable development goals in G-10 economies. Increasing energy productivity in the G-10 economies will not only increase economic output but also contribute to the management of energy demand. In addition, energy productivity in the G-10 economies is a critical factor in achieving carbon neutrality. The results of this study are comparable to those of [13,18,94]. However [18], reported that energy productivity and eco-innovation decrease G-7 economies' carbon emissions. While [18] worked on the G-7 economies to investigate the impact of energy productivity on carbon emissions, they found that increasing energy production reduces carbon dioxide emissions and improves environmental quality. In this context [94], discovered that energy productivity considerably decreases CO_2_ emissions in an empirical analysis of the relationship between eco-innovation, energy productivity, and carbon emissions. Moreover, according to their research, eco-innovation reduces carbon emissions in G-7 economies. Similarly [95], analyzed the link between energy output and energy consumption in OECD countries between 1990 and 2017. Their research revealed a correlation between a nation's energy production and its energy consumption pattern. Prior research on the connection between energy output and CO_2_ emissions has primarily focused on industrialized countries. Thus, research examining the impact of energy production on CO_2_ emissions for the N-11 economies is crucial, as they are developing countries experiencing rapid economic growth.

The coefficient of renewable electricity generation is −0.6920% long run and −0.6471% short run, indicating that a 1% increase in ENP is recorded to reduce CO_2_ emissions by 0.6920% and 0.6471% in the long and short-run estimations, respectively. However, the long-run influence of renewable electricity generation is moderately bigger than the short-run effect. The finding is consistent with the finding of [96], It is crucial for the countries under study to achieve carbon zero while protecting a healthy ecosystem, recognizing renewable electricity generation as a productive element to reduce excessive carbon output, and strongly encouraging the use of renewable sources over non-renewable energy sources. Moreover, employing ecologically destructive energy sources such as fossil fuels has had a substantial influence on the environment throughout history [59,93,97].

The coefficient of ETX is −0.0547 and −0.0785 in the long run and short run, indicating that a 1% increase in REG is recorded to reduce CO_2_ emissions by 0.0547% and 0.0785% in the long and short-run estimations, respectively. However, the long-run influence of ETX is moderately bigger than the short-run effect. Thus, environmental taxes provide a variety of advantages: (a) Environmental impacts can be reduced by reducing energy consumption, which reduces emissions and fossil fuel consumption; (b) negative externalities can be corrected, for instance, by implementing a carbon tax; (c) energy security can be improved by increasing domestic energy production and promoting renewables, which reduces reliance on imported energy; and (d) carbon taxes can increase government income, which can be recycled for other reasons. It is intended that by imposing carbon taxes, fuel-intensive items would be substituted, resulting in a shift in the structure of energy production and consumption towards more environmentally friendly products [98]. Apart from promoting cleaner energy production and consumption, which improves environmental quality, environmental taxes, as proposed by the “double dividend” hypothesis, have the potential to raise funds for governments, with the possibility of recycling these funds to correct other economic distortions. Environmental tax revenues, for example, can be used to cut a distortionary tax (such as wages) and eliminate current economic inefficiencies [84]. At the 10% level of significance, the negative value of the ECM coefficient by −0.17141. According to these results, the model's annual adjustment to long-run equilibrium is about 17.2%. Theoretical predictions and analysis support negative findings about ECM.

This study also used FMOLS and DOLS to assess how reliable the *C*S-ARDL approach's long-term estimates were, and Table 8 presents the results of these two tests. As per the findings, in the long run TEC, ECI, ENP, REG, and ETX have negative effects on CO_2_ emissions. These studies produced results that were exactly the same as those from earlier ones, proving the reliability and consistency of the *C*S-ARDL approach's findings. The outcomes of the econometric analysis performed using the AMG method also indicate that variables might have an effect on CO_2_ emissions in the G-10 economies. These findings are reliable and compatible with the conclusions obtained using the *C*S-ARDL method.

**Table 8:** Robustness checks.

Variable	FMOLS		DOLS		AMG	
	Coeff.	Prob.	Coeff.	Prob.	Coeff.	Prob.
TEC	−0.6785*	0.0651	−0.0717*	0.0003	0.9710*	0.000
ECI	−0.5177*	0.0000	−0.6082*	0.0005	−0.6223*	0.000
ENP	−0.1795*	0.0000	−0.2099**	0.0324	−0.3514*	0.000
REG	−0.3793*	0.0000	−0.3978*	0.0010	−0.4521*	0.000
ETX	−0.2987*	0.0001	−0.1578*	0.0000	−0.2141*	0.000

Note: * and ** designate the significance level at 1%, and 5% respectively.

Source: Authors' estimation.

The estimates of the D-H Granger causality test are presented in Table 9. The findings exhibit that a one-way causal linkage was exposed from CO_2_ to REG, CO_2_ to ETX, REG to TEC, ENP to ECI, REG to ECI, ETX to ECI, ETX to ENP and ETX to REG. While bidirectional causality exists between TEC and CO_2_, ECI and CO_2_, ENP and CO_2_, REG and ENP. Finally, there is no causality exist between ECI and TEC, ENP and TEC, ETX and TEC.

**Table 9:** Granger causality.

Null Hypothesis:	W-Stat.	Z-Stat.	Prob.	Remarks
TEC ⇎ CO_2_	6.0296*	4.7694	0.0000	TEC ⇐⇒ CO_2_
CO_2_ ⇎ TEC	4.8442*	3.2794	0.0010	
ECI ⇎ CO_2_	5.8409*	4.5323	0.0000	ECI ⇐⇒ CO_2_
CO_2_ ⇎ ECI	4.5968*	2.9684	0.0030	
ENP ⇎ CO_2_	5.9956*	4.7267	0.0000	ENP ⇐⇒ CO_2_
CO_2_ ⇎ ENP	4.9121*	3.3647	0.0008	
REG ⇎ CO_2_	3.3280*	1.3735	0.0009	CO_2_ ⇒ REG
CO_2_ ⇎ REG	4.5503*	2.9100	0.0036	
ETX ⇎ CO_2_	2.2149**	0.5381	0.0790	CO_2_ ⇒ ETX
CO_2_ ⇎ ETX	5.3301*	3.8902	0.0001	
ECI ⇎ TEC	2.5366*	0.3787	0.0080	ECI ⇎ TEC
TEC ⇎ ECI	1.6621	−0.7206	0.4711	
ENP ⇎ TEC	2.5366	0.3787	0.0704	ENP ⇎ TEC
TEC ⇎ ENP	2.0541	−0.2277	0.3760	
REG ⇎ TEC	1.6817	−0.6958	0.4865	REG ⇒ TEC
TEC ⇎ REG	5.5273*	4.1380	0.0000	
ETX ⇎ TEC	3.3703	1.4267	0.0421	ETX ⇎ TEC
TEC ⇎ ETX	3.1150	1.1057	0.0531	
ENP ⇎ ECI	1.6620	−0.7206	0.3111	ENP ⇒ ECI
ECI ⇎ ENP	2.0541	−0.2277	0.5199	
REG ⇎ ECI	4.4721*	3.4088	0.0007	REG ⇒ ECI
ECI ⇎ REG	3.7447**	1.8974	0.0578	
ETX ⇎ ECI	5.3262*	3.8852	0.0001	ETX ⇒ ECI
ECI ⇎ ETX	2.9116*	0.8501	0.3952	
REG ⇎ ENP	7.0803*	6.0902	0.0000	REG ⇐⇒ ENP
ENP ⇎ REG	5.6277*	4.2643	0.0000	
ETX ⇎ ENP	5.5402*	4.1543	0.0000	ETX ⇒ ENP
ENP ⇎ ETX	2.8623	0.7882	0.2306	
ETX ⇎ REG	3.8574*	2.0390	0.0414	ETX ⇒ REG
REG ⇎ ETX	3.4027	1.4675	0.0622	

Note: * and ** designate the significance level at 1%, and 5% respectively.

Source: Authors' estimation.

## 6 Conclusion and policy recommendation

### 6.1 Conclusion

The purpose of this study is to assess the influence of the economic complexity index, energy productivity, renewable electricity generation, and environmental taxes on CO_2_ emissions in the G-10 countries from 1995 to 2020. To address CD and heterogeneity, this research employed a second-generation econometrics method. In this research, we employed the CIPS and CADF unit root tests, the *C*S-ARDL, the D-H Granger causality test, and the FMOLS, DOLS, and AMG estimators for robustness analysis. Technology, economic complexity, renewable electricity generation, energy productivity, and environmental taxes all have a negative influence on CO_2_ emissions, as reported by the *C*S-ARDL, FMOLS, DOLS, and AMG tests and validated by the CIPS and CADF tests, respectively. Estimates based on the D-H. Granger causality between TEC and CO_2_, ECI and CO_2_, and ENP and CO_2_ all pointed in the same direction, suggesting a bidirectional causal relationship. The adoption of renewable energy sources that lessen reliance on fossil fuels was therefore suggested as a means to achieve a low-carbon future. It is essential to increase energy output for a sustainable environment, and the best ways to do so are through the promotion of renewable energy supply and the improvement of energy efficiency. To support the creation of ecologically beneficial technology and concepts based on renewable energy sources, governments must execute broad-scale government policies and initiatives.

### 6.2 Policy recommendations

Environmental technology, economic complexity, energy productivity, the use of renewable energy to generate electricity, and environmental taxes on CO_2_ emissions in the G-10 countries are all factors that can be addressed with policy recommendations based on the findings of this study to help ensure a sustainable and low-carbon future. Increase investment in research and development of clean energy technologies to decrease the costs of renewable energy and they should develop and implement policies to promote renewable energy, such as solar, wind, hydro, geothermal, and biomass, and energy efficiency in order to reduce reliance on fossil fuels and decrease carbon emissions.

It is imperative that the G-10 create and execute progressive environmental tax policies to encourage carbon reduction and deter the use of fossil fuels. Implementing carbon taxes, cap-and-trade programs, and other mechanisms that put a price on carbon emissions and stimulate private sector finance for SDGs by providing incentives and laws that promote investment in sustainable and low-carbon projects are all examples of such methods. To speed up the shift to a sustainable and low-carbon future, the G-10 countries should encourage international cooperation to exchange knowledge, resources, and technologies. It may involve helping those nations improve their use of renewable energy sources, increase energy efficiency, and safeguard the environment. To sum up, a sustainable and low-carbon future in the G-10 countries requires an all-encompassing policy strategy that incorporates environmental protection, economic complexity, renewable energy usage to generate electricity, energy productivity, and environmental taxes on CO_2_ emissions.

The G-10 nations should prioritize increasing energy efficiency in houses, vehicles, and industries in addition to promoting the utilization of alternative and renewable energy sources. Building rules, energy efficiency standards for appliances and equipment, and public awareness campaigns can all help make this a reality. Since climate change affects the entire planet, the G-10 should collaborate to share ideas and work towards a low-carbon, sustainable future. The Paris Agreement, the G-7 Energy Ministerial, and the International Renewable Energy Agency are all examples of possible initiatives that could be used for this purpose. This list is not comprehensive, and the right balance of policies for a given country may vary based on its unique characteristics and available resources. Nonetheless, many specialists think that these policies, when combined, can pave the way for a low-carbon future in the G-10 nations.

### 6.3 Constraints opportunities for future and research

This study looked at the effects of environmental technological innovation, economic complexity, energy productivity, the use of renewable electricity generation, and environmental taxes on CO_2_ emissions in the G-10 countries. Other scholars can add to the EKC argument by looking at covariates like population and urbanization in an asymmetric framework for other blocs such as the ASEAN, OECD, BRICS, G-7, E−7, and others. This study's scope can be expanded by examining the effects of environmental technology innovation, economic complexity, energy productivity, the usage of renewable electricity generation, environmental levies on CO_2_ emissions, an ecological footprint, and other proxies of environmental pollution. It is also conceivable to do a country-by-country and industry-by-industry analysis in order to reconsider and reformulate policymaking objectives. The availability of data is also one of the core issues of this study. In this regard, the upcoming studies should use some other variables (as proxies) to address this caveat. Carbon emissions in the G-10 nations can be analyzed through the indirect effects of environmental technological progress and renewable electricity production.

## Data availability statement

The open-access datasets employed in the analyses can be accessed from the following links: https://databank.worldbank.org/source/world-development-indicators (accessed on December 09, 2022) and https://data.oecd.org/searchresults/?q=indicators (accessed on December 16, 2022).

## Funding

This research received no external funding.

## Author contribution statement

Najia Saqib; Magdalena Radulescu: Conceived and designed the experiments; Performed the experiments; Analyzed and interpreted the data; Wrote the paper.

Muhammad Usman: Performed the experiments; Analyzed and interpreted the data; Contributed reagents, materials, analysis tools or data; Wrote the paper.

Daniel Balsalobre-Lorente; Teodor Cilan: Analyzed and interpreted the data; Contributed reagents, materials, analysis tools or data; Wrote the paper.

## Additional information

No additional information is available for this paper.

## Declaration of competing interest

The authors declare that they have no known competing financial interests or personal relationships that could have appeared to influence the work reported in this paper
